# A Painful Immobile Shoulder

**DOI:** 10.1016/j.acepjo.2026.100423

**Published:** 2026-05-27

**Authors:** Niklas Damberg, Wesley Eilbert

**Affiliations:** Department of Emergency Medicine, University of Illinois, College of Medicine Room, Chicago, Illinois, USA

**Keywords:** luxation erecta, inferior shoulder dislocation, luxation erecta humeri

## 1 Patient Presentation

**Figure 1:**
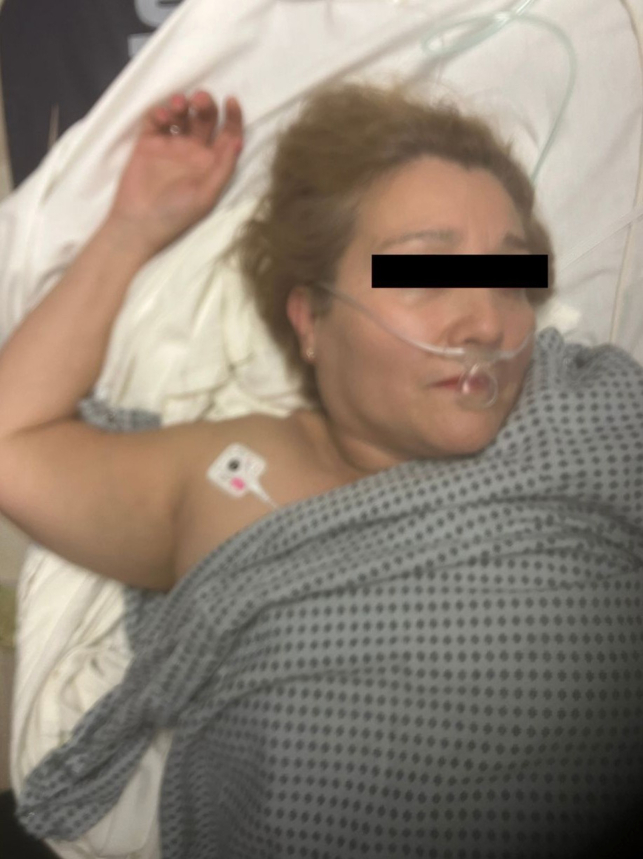
Arm hyperabducted and locked above the patient’s head.

**Figure 2:**
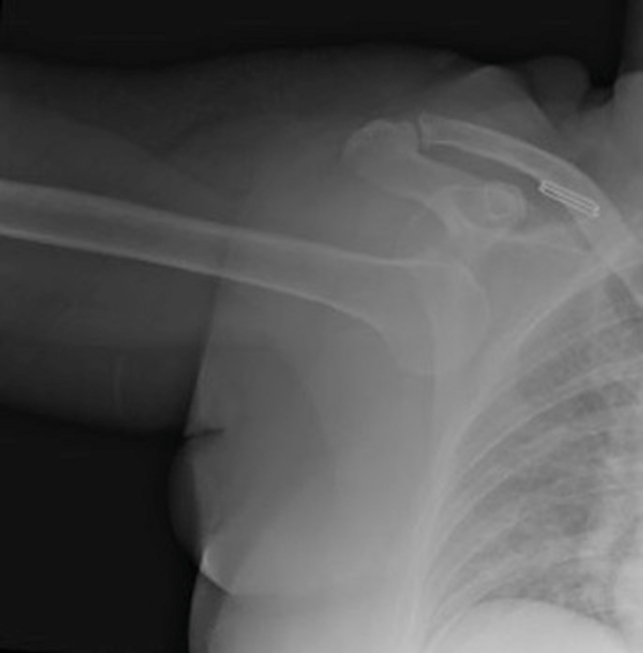
Anteroposterior view of the right shoulder showing inferior dislocation.

A 54-year-old woman presented to the emergency department, complaining of right shoulder pain with inability to move her right arm from an overhead position (Fig 1). She reported falling forward while attempting to open a window, resulting in an axial load onto her abducted arm, causing the injury. A radiograph of her right shoulder was obtained (Fig 2).

## 2 Diagnosis

### 2.1 Luxatio erecta humeri

First described in 1859, luxio erecta humeri (LEH) is a rare inferior shoulder dislocation, accounting for only 0.5% of all shoulder dislocations.^1^^,^^2^ More common in males and occurring in all age groups, LEH typically occurs as the result of a fall causing hyperabduction of the shoulder or axial loading on an abducted arm.^3^^,^^4^ As with this case, patients classically present with shoulder pain and their arm hyperabducted and locked above their head.^5^

Associated bony injuries are common with LEH, with 39% having a fracture of the proximal humerus, most commonly the greater tuberosity.^6^ Up to 59% of LEH will have evidence of a peripheral nerve injury, most commonly a neuropraxia, and typically involving the axillary nerve.^3^^,^^5^^,^^6^ Vascular injuries occur in up to 10% of LEH, most often involving the axillary artery.^3^^,^^6^ Approximately half of LEH cases will have tears of the rotator cuff.^5^ As with this patient, the vast majority of LEH can be reduced in a closed manner with a traction counter traction maneuver.^5^ Following reduction, shoulder immobilization with a sling adducted to the chest is indicated.^5^

## Funding and Support

By *JACEP Open* policy, all authors are required to disclose any and all commercial, financial, and other relationships in any way related to the subject of this article as per ICMJE conflict of interest guidelines (see www.icmje.org). The authors have stated that no such relationships exist.

## Conflict of Interest

All authors have affirmed they have no conflicts of interest to declare.
